# Torsion of vermiform appendix with fecalith: a case report

**DOI:** 10.1186/1757-1626-1-20

**Published:** 2008-06-23

**Authors:** Imtiaz Wani, Maki Kitagawa, Mudasir Rather, Jang Singh, Gulam Bhat, Mir Nazir

**Affiliations:** 1 Department of Surgery, S.M.H.S Hospital, Srinagar, India; 2 Department of Surgery, Shakaihoken Kobe Central Hospital, Kobe, Japan; 3 Department of Pathology, S.M.H.S Hospital, Srinagar, India

## Abstract

**Introduction:**

Torsion of the vermiform appendix is a rare disorder, which causes abdominal symptoms indistinguishable from acute appendicitis and is found by chance during the laparotomy.

**Case presentation:**

We report a case (a 76-year-old male) suffering of torsion of the vermiform appendix with fecalith. It was twisted 540 degrees in an anti-clockwise direction. Appendectomy was done.

**Conclusion:**

Appendiceal torsion may be assocated with the presence of fecalith. This case is the oldest one among the patients with appendiceal torsion reported to literature.

## Introduction

Torsion of the vermiform appendix is a rare disorder, which causes abdominal symptoms indistinguishable from acute appendicitis. Primary and secondary torsion are recognized in the appendiceal torsion. In primary torsion, specimen examination shows secondary ischemic or necrotic change and luminal dilatation distal to the torsion site without any primary lesion. About 25 cases of primary torsion have been reported to date. Secondary torsion is much rarer and only 7 cases have been reported to date. Secondary torsion is caused by appendiceal abnormality, which includes cystadenoma, mucocele, fecalith impaction, and malformation [1-11]. Preoperative diagnosis of torsion of the appendix is sometimes difficult. Here, we report a case of appendiceal torsion with fecalith. This case (a 76-year-old male) is the oldest one among the patients with appendiceal torsion reported to date.

## Case presentation

**Figure 1:**
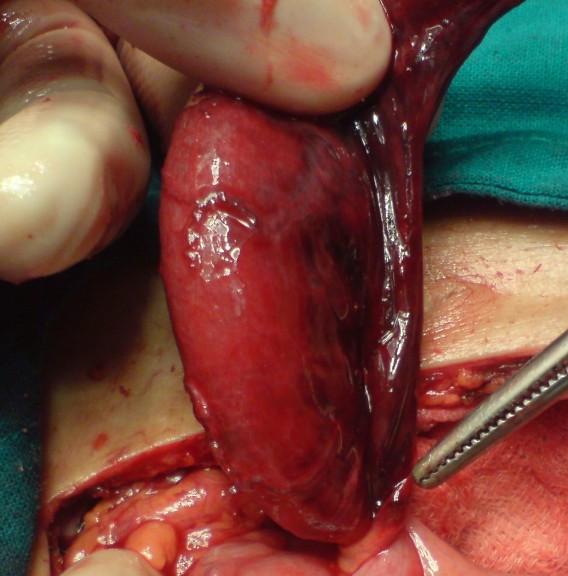
The appendix was twisted 540° in an anti-clockwise direction at the point of 1 cm distal to its base.

**Figure 2:**
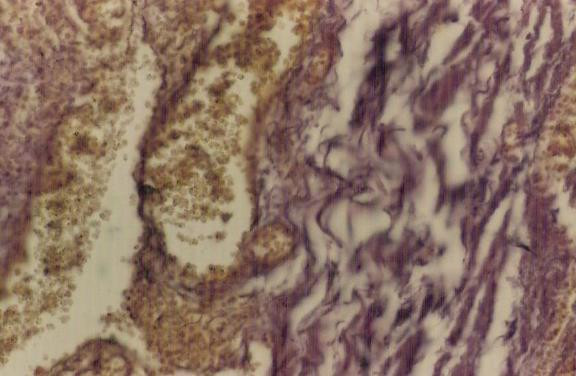
Histopathological examination of specimen showing mild inflammation, prominent congestion of mucosal and submucosal vascular channels and intraluminal haemorrhage within appendix.

A 76-year-old male had upper abdominal pain early in the morning. Four hours after the initial attack, he had severe pain in the right lower quadrant area, which was accompanied with nausea. On physical examination, his body temperature was 99.0°F, pulse 98 beats/min, and blood pressure 130/80 mmHg. There was tenderness and rebound tenderness in the right lower quadrant area, and positive Rovsing's sign. Peripheral blood examination showed a white blood cell count of 11,000/mm^3 ^(78% neutrophils). Abdominal X-ray examination revealed small-intestinal dilatation with air-fluid level. Modified Alvarado's score for appendicitis was nine points. Laparotomy was done on the suspected diagnosis of acute appendicitis. The appendix was 10 cm in length and 1.5 cm in diameter, and showed gangrenous change. It was twisted 540 degrees in an anti-clockwise direction at the point of 1 cm distal to its base (Fig. 1). A simple appendectomy was performed. The appendix was filled with mucous and contained fecalith in it. Postoperative recovery was uneventful. Histological examination of the specimen revealed that there was mild inflammation with some hemorrhage and necrosis in the appendiceal wall, the findings are consistent with the ischemic and necrotic changes caused by the torsion (Fig. 2).

## Discussion

In 1918, Payne et al [12] reported the first case of torsion of the appendix. Torsion of the appendix may occur at any age (range 3–60 years). No case of more than 60 years old has been reported. Our case was 76 years old, and, thus, was the oldest one among the patients with appendiceal torsion reported to date [1] Primary torsion appears to be often associated with long appendix. The appendix averages 6.5 cm (ranging from 1 to 20 cm) in length. Secondary torsion has been reported to be associated with cystadenoma, mucocele, fecalith impaction, and malformation of the appendix. The presence of fecalith has been also reported to be closely associated with acute appendicitis [13]. Torsion of the appendix observed in this case may be, at least in part, caused by the presence of fecalith, because no other primary lesions and malformation were detected. The preoperative diagnosis of appendiceal torsion is sometimes difficult. Abdominal computed tomography (CT) examination with the contrast medium has been shown to be helpful for the preoperative diagnosis of appendiceal torsion [1]

## Conclusion

Torsion of the vermiform appendix is a rare disorder, which causes abdominal symptoms indistinguishable from acute appendicitis. Our case (a 76 year-old male) was the oldest one among the patients with appendiceal torsion reported to date. In this case, the presence of fecalith may be associated with torsion of the appendix.

## Competing interests

The authors declare that they have no competing interests.

## Authors' contributions

IW was involved in the acquisition of data, compilation of relevant literature and drafted the preliminary and final manuscript.

MK was involved with the revision, formatting and proofreading the manuscript.

MR carried out the operation of this patient and helped to draft the manuscript.

JS prepared the histopathological photographs for the manuscript.

GB supervised the project and reviewed the manuscript.

MN undertook final revision of manuscript and was done under his supervision.

All authors read and approved the final version of manuscript.

## Consent

Written informed consent was obtained from the patient for publication of this case report and accompanying images. A copy of the written consent is available for review by the Editor-in-Chief of this journal
